# Preclinical pharmacokinetics, distribution, metabolism and excretion of disitamab vedotin

**DOI:** 10.5599/admet.2582

**Published:** 2025-03-14

**Authors:** Ling Wang, Limeng Zhu, Fengzhu Wang, Lihou Dong, Zhihao Liu, Fang Chen, Jing Jiang

**Affiliations:** 1 RemeGen Co., Ltd, Yantai 264000, Shandong, China; 2 Rongchang Industry College, 264003, Shandong, China; 3 New York University, Grossman School of Medicine, New York, NY, USA; 4 United-Power Pharma Tech Co. Ltd, Beijing 102206, China; 5 Department of Pharmacology, Binzhou Medical University, Yantai 264003, Shandong, China

**Keywords:** Antibody-drug conjugate, physiological disposition, monomethyl auristatin E (MMAE), mass balance

## Abstract

**Background and purpose:**

Disitamab vedotin is an antibody-drug conjugate (ADC) composed of a humanized IgG1 monoclonal antibody (mAb) targeting HER2 conjugated to monomethyl auristatin E(MMAE) via a cleavable dipeptide linker.

**Experimental approach:**

The pharmacokinetics, distribution, catabolism/metabolism and elimination properties of disitamab vedotin and its payload MMAE were characterized in rats and tumour-bearing mice.

**Key results:**

The configured mAb and total antibody showed linear dynamic characteristics. Moreover, the molecular structure of disitamab vedotin effectively reduces the exposure of MMAE, which has a fast clearance. Two radiolabeled probes were developed to track the fate of different components of the disitamab vedotin, including ^125^I labelled antibody and ^3^H labelled MMAE payload of the ADC. Following a single intravenous administration of the radiolabeled probes to the tumour-bearing mice and rats, blood, various tissues, and excreta samples were collected and analyzed for radioactivity and to characterize the metabolites/catabolites. Disitamab vedotin and free MMAE (FM) were majorly distributed in tissues and organs with rich blood flow. Moreover, both disitamab vedotin and MMAE have higher and longer exposure in tumour tissue. Disitamab vedotin was mainly eliminated through renal excretion, while the FM was mainly eliminated through the biliary faecal route (>70 %) and a small fraction (<10 %) was eliminated through renal excretion in the form of catabolites/metabolites, among which, MMAE was identified as the major species, along with 10 other minor species.

**Conclusion:**

These studies provided significant insight into disitamab vedotin pharmacokinetics, distribution, metabolism and elimination properties, which supports the clinical development of disitamab vedotin.

## Introduction

Antibody-conjugated drugs (ADC) are effective antitumor drugs conjugated by monoclonal antibodies and small molecule cytotoxic loads through a linker. ADCs are altering the modern oncology landscape, possessing both the high targeting performance of monoclonal antibodies and the powerful killing effect of small molecule cytotoxic drugs [1]. Meanwhile, the global sales of currently marketed ADCs are forecast to exceed $16.4 billion in 2026 [2]. Therefore, researchers have continuously increased their enthusiasm for ADC in recent years. As of February 2024, fifteen ADCs have been approved for marketing worldwide, and about 260 are in clinical studies. Disitamab vedotin (DV), developed by RemeGen. Co., Ltd, was approved by the National Medical Products Administration (NMPA) for the treatment of advanced gastric cancer in 2021. DV also received Food and Drug Administration Investigational New Drug (FDA IND) approval for gastric cancer or gastroesophageal junction cancer in 2020 and was awarded breakthrough therapy designation for urothelial cancer by the FDA in the same year [3]. DV, an ADC composed of a humanized IgG1 mAb (Hertuzumab) targeting HER2 conjugated to a microtubule-disrupting drug MMAE via a cleavable dipeptide linker. DV showed superior antitumor activity than T-DM1 in trastuzumab- and lapatinib-resistant xenograft tumour models, suggesting its potential as an improved therapy for HER2-positive breast cancers [7-8].

The absorption, distribution, metabolism, and excretion (ADME) studies for therapeutic biologics could be a challenging task due to the complex natures (*e.g.* ADC conjugate, TA, and unconjugated cytotoxic drug) of these novel molecules and limited bioanalytical methods [9]. Nevertheless, knowledge gained from in-depth studies of nonclinical and clinical ADME mechanistics will advance our understanding of the in vivo disposition of various therapeutic biologics. In addition, ADME knowledge contributes to understanding clinical toxicity, optimizing dispositional properties, improving human efficacy, and the safety of new therapeutic biologics [10]. As a result, a deep and thorough understanding of the preclinical ADME properties of ADC drugs is full of challenges but very meaningful.

Preclinical ADME is usually performed in rodents, and radio labelling techniques are used in tissue distribution, metabolism and excretion experiments [14]. In this paper, we carried out the PK, distribution, excretion (^125^I/^3^H labelled), and metabolism preclinical study in rodents to illustrate the pharmacokinetics, metabolite, and disposition characteristic of DV. to provide more theoretical basis and data support for clinical. The ADME studies have provided more theoretical basis and data support for clinical and will provide references for future ADC development.

## Materials and methods

### Antibodies and antibody-conjugated drugs

Disitamab vedotin (DV) was generated by RemeGen Co., Ltd., as described previously [3]. The average drug-antibody ratio (DAR) was 3.5-4.5 MMAE per mAb.

### Radiochemistry

For [^125^I] radiolabeling, Iodogen (Sigma-Aldrich, St. Louis, MO, USA) was added to the test tube and, subsequently, conjugated to the DV (RemeGen, YanTai, CN). Then, to remove the excess unconjugated [^125^I], the solution was passed through a 1×50 cm SEPHADEX G10 column (Pharmacia, New York, USA). The radiolabeling DV was characterized by radiochemical purity, protein content, and bioactivity.

**Figure 1.**
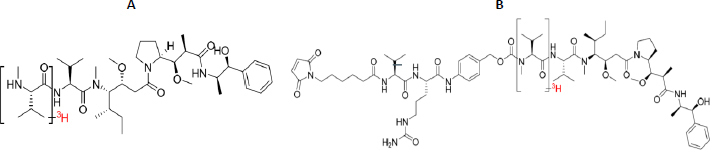
Structure of [^3^H] MMAE (A) and MC-Val-Cit-PAB-C-[^3^H] MMAE(B)

The [^3^H] MMAE (lot: K0903) and MC-Val-Cit-PAB-C-[^3^H] MMAE (lot: K0904) were obtained from Curachem Inc. (Chungcheongbuk-do, South Korea), with a specific activity of 17.57 Ci/mmol. MC-Val-Cit-PAB-C-[^3^H] MMAE with the specific activity of 17.57 Ci/mmol (Curachem Inc., Chungcheongbuk-do, South Korea) was conjugating to RC48-ADC mAb, as described in the above previously Error! Reference source not found.. The resulting [^3^H-MMAE]-RC48-ADC had a specific activity of 254 μCi/mL and a DAR of 3.96. The structure of [^3^H] MMAE and MC-Val-Cit-PAB-C-[^3^H] MMAE are shown in Figure 1A and Figure 1B, respectively.

### Animal housing and procedure

All animals in the study were purchased from qualified Laboratory Animal Technology Co., LTD (production license number: 2018-0001, 2016-0011, 2012-002, 2012-0004) and housed in an SPF environment. Bile-duct-cannulated (BDC) rats were obtained from Charles River Laboratories (Beijing, CN) after surgery and recovery. The mice were subcutaneously inoculated with human gastric cancer NCI-N87 cells. Follow-up experiments were carried out after the tumours had grown to 300 to 400 mm^3^.

All animal studies were carried out in accordance with the requirements of the Association for Assessment and Accreditation of Laboratory Animal Care International. All the experimental protocols have been reviewed and approved by the Institutional Animal Care and Use Committee (IACUC) before implementation.

### PK profile of RC48-ADC and MMAE in rats

Twenty-four 50/50 male and female rats, 8 to 12 weeks old, weighing approximately 200 g, were administered RC48 (4, 8, 16 mg/kg) or RC48 bare Ab in combination with MMAE (8 mg/kg RC48 Ab and 0.16 mg/kg MMAE) in a single i.v. dose. Blood samples were collected at 0min (Predose), 1, 10 and 30 min, 2 and 8 h, then 1, 2, 3, 6, 8, 10, 12, 14, 17, 21, 24 and 28 days after administration, followed by centrifugation (4 °C, 1000 to 2000 g, 10 min) to separate plasma. The upper serum was divided into three fractions and analysed after freezing at -80 °C. Concentrations of coupled antibodies (ADC), total or naked antibodies, and free MMAE were determined for each of the three fractions of serum samples.

### Tissue distribution study with ^125^I-RC48-ADC

The tumour-bearing nude mice were killed by femoral artery bleeding at 4, 24, 72 and 168 h after i.v. injection of 12 mg/kg ^125^I-RC48-ADC. Samples from the following 23 tissues/body fluids were collected, including urine, serum, brain, tumour tissue, and so on. The total gamma radioactivity was measured in each tissue. An equal volume of 20 % trichloroacetic acid (TCA) was added to precipitate the proteins (final concentration = 10 %). The gamma radioactivity in the TCA precipitate of each tissue was measured after the samples were centrifuged and the supernatant was removed. The radioactive concentration in tissue/body fluid was expressed as μg equivalents per gram of fresh wet weight (μg·eqv·g^-1^) for tissues or per mL for body fluids (μg·eqv·mL^-1^). Radioactivity was investigated using the size-exclusion high-performance liquid chromatography (SHPLC) and radioactive analysis. The Radiomatic^™^ 610TR Flow Scintillation Analyzer (Packard Instrument Co.) was used to scan the gamma radioactive chromatograms. The peaks were directly analysed by the FLO-ONE software for Windows from the same company.

### Tissue distribution study with [^3^H-MMAE]-RC48-ADC

The NCI-N87 tumour-bearing nude mice were killed by femoral artery bleeding and twenty-five tissues/body fluids were collected at 4, 24, 72, 168 and 336 h after i.v. injection of 12 mg/kg [^3^H-MMAE]--RC48-ADC. All these tissues/body fluids included plasma, tumour, heart, spleen, lung, kidney and liver homogenates, extracted with 6 times the volume of acetonitrile (ACN) and centrifuged at 10,000*g* for 10 min. The concentration of free MMAE was obtained by direct measurement of all acetonitrile extracts. After centrifugal precipitation, whole blood and remaining tissue specimens were dissolved by heating in a 1 M KOH solution at 90 °C and the radioactivity content was determined by liquid scintillation counting. Radioactivity concentrations in tissues/body fluids were expressed as microgram equivalents per gram of fresh wet weight (μg·eqv·g^-1^) or per millilitre of body fluid (μg·eqv·mL^-1^). Total radioactivity and ACN extract radioactivity (^3^H-labelled MMAE) were determined using Radioactive Liquid Scintillation Analyzer.

### ^125^I-RC48-ADC and [^3^H-MMAE]-RC48-ADC excretion study

Urine and faecal samples were collected from 0 to 24 h, 24 to 48 h, ..., to 288 to 336 h after intravenous injection of 12 mg /kg ^125^I-RC48-ADC in twelve male and female tumour-bearing nude mice. Urine and faecal samples also were collected at 0 to 8 h, 8 to 24 h, …, until 336 to 504 h after intravenous administration of 8 mg/kg [^3^H-MMAE]-RC48-ADC to six intact rats of both sexes. The gamma radioactivity per unit volume was measured. The amount of the radioactivity excreted as a percentage of the administered radioactivity was calculated.

The abdominal wall of rats was dissected under anaesthesia and sodium pentobarbital-assisted anaesthesia, and the common bile duct was isolated. The distal end of the bile duct was ligated, and a drain was inserted at the proximal end to drain the bile. Each rat received a dose of 8 mg/kg ^125^I-RC48-ADC or [^3^H-MMAE]-RC48-ADC via intravenous injection on the tail. Bile samples were collected at 1, 2, 3, 5, 8, 12 and 24 h or 4, 8, 24, 48 and 72 h after the dose. The gamma radioactivity per hour in the bile was measured. The 24-hour cumulative excreted radioactivity was calculated as a percentage of the total administered radioactivity and the changes over time were examined.

### Catabolite profiling and identification with [^3^H]-MMAE disitamab vedotin in rats

The bile urine and faecal homogenate samples used for metabolic research in this study were all from the excretion studies of rats after a single intravenous administration of 8 mg/200 Ci/kg [^3^H-MMAE] RC48-ADC, and the plasma and tumour tissue homogenate samples were from a single intravenous administration of 12 mg/200 of tumour-bearing mice tissue distribution after [^3^H-MMAE]RC48-ADC of Ci/kg.

To characterize the catabolic profile of [^3^H]-MMAE DV conjugated with plasma, bile, urine, faeces, and tumour tissue, samples were investigated using a Shimadzu LC-2030C 3D (Shimadzu Company, JP) coupled with an LTQ Orbitrap XL System (Thermo Scientific, USA) and an online-Model 3 radio detector (America In Motion, USA) for radio profiling. The significant radioactive metabolites and structure of metabolites were identified and analysed by LC/RAM/MS method. The structural analysis of all metabolites was based on the metabolic pathway and relative retention time of HPLC.

Chromatographic separation was performed on a C18 column (150×4.6 mm, 3.0 μm particle size, ACE, UK) with mobile phases A (0.4 % formic acid in the water was adjusted to pH 3.2 with NH_4_OH) and B (acetonitrile) at a constant flow rate of 0.7 mL/min. The gradient was as follows: initial holding at 0 % B for 3 min, increased to 35 % B at 30 min, holding at 35 % B until 45 min, 100 % B at 55 min, holding at 100 % B until 58 min, decreasing to 0 % B at 60 min, and then column re-equilibration until 72 min. The flow was split 4:1 post-column for the radio measurements and mass spectrometry.

## Results and discussion

### Pharmacokinetic profile of RC48-ADC and MMAE in rats

**Figure 2.**
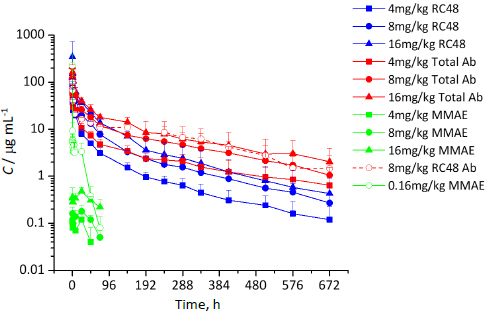
Serum concentration−time profiles of RC48-ADC, total Ab, and MMAE in rats after iv administration of RC48-ADC(4 to 16 mg/kg) or RC48-ADC Ab combination with MMAE (8+0.16 mg/kg) (Mean ± SD, *n* = 6 per time point)

The mean serum concentration-time curves of total antibody (TA), conjugated antibody (CA), free MMAE (FM) in rats in different administration groups are shown in Figure 2. The corresponding pharmacokinetics (PK) parameters of TA, CA and FM are presented in Table 1. After a single iv administration of RC48-ADC at 4 to 16 mg/kg in Rats, the exposure levels of CA were positively correlated with the dose (Figure 2 and Table 1), and the half-life (*T*_1/2_ - time required for the concentration of a drug or a substance in the body to decrease by half.), clearance (CL - volume of blood or plasma that is cleared of a drug per unit time) and mean residence time to infinity (MRT_inf_ - average time that a drug molecule spends in the body from the time of administration until it is completely eliminated) were consistent, showing linear dynamic characteristics. T-test results illustrated no significant differences in *T*_1/2_, time to maximum concentration (*T*_max_ - time it takes for a drug to reach its maximum concentration in the bloodstream or a specific tissue after administration), CL and MRT_inf_ of CA between the different dose groups (*P*>0.05), showing linear dynamics characteristics. At the same administration dose, the AUC of CA was significantly lower than that of TA (*P*<0.05), and the system clearance rate of CL was significantly higher than that of TA (*P*<0.05), indicating that the clearance of CA in vivo was greater than that of Tab (Table 1). As a result, they exhibited approximately the same half-lives, while the generally shorter clearance was observed for Tab (Table 1). In addition, the corresponding systemic exposures showed a positive correlation with the dose escalation from 4 to 16 mg/kg (Table 1).

**Table 1.** PK parameters of RC48-ADC, total Ab and MMAE following iv administration of RC48-ADC (4 to16 mg/kg) or RC48-ADC Ab combination with MMAE (8 + 0.16 mg/kg) to rats.

Parameter		*T*_1/2_ / h	*T*_max_ / h	*C*_max_^a^ / μg mL^-1^	AUC_(0-t)_^b^, h μg mL^-1^	AUC_(0-inf)_^b^, h μg mL^-1^	AUC_(t-inf)_, %	*V*_d_ / mL kg^-1^	CL, (mL h^-1^) kg^-1^	MRT_inf_, h
RC48-ADC 4 mg kg^-1^	Total Ab	206.94± 83.34	0.02±0.00	63.18±8.23	1974.28±463.83	2229.21±613.9	10.64±5.62	534.62±165.47	1.90±0.47	240.13±79.75
	RC48-ADC	180.28±82.44	0.02±0.00	59.07±12.22	1096.53±115.05	1150.01±134.65	4.51±2.91	894.72±347.88	3.52±0.41	133.88±45.07
	MMAE	-	12.34±11.66	0.137±0.029	4.15±1.05	-	-	-	-	-
RC48-ADC 8 mg kg^-1^	Total Ab	184.21±48.56	0.04±0.06	120.88±30.79	4561.78±375.70	4891.05±410.53	6.70±1.86	434.91±119.47	1.65±0.14	208.51±44.18
	RC48-ADC	174.08±52.23	0.04±0.06	96.67±19.42	2541.41±195.97	2634.57±208.01	3.52±1.47	768.59±249.79	3.05±0.25	127.32±26.48
	MMAE	-	17.34±14.66	0.22±0.039	8.87±1.15	-	-	-	-	-
RC48-ADC 16 mg kg^-1^	Total Ab	156.86±114.43	0.10±0.20	214.4±19.86	6793.91±2389.51	7547.62±2840.88	9.07±6.62	443.65±248.72	2.42±1.00	220.43±107.77
	RC48-ADC	117.17±73.81	0.02±0.00	344.5±395.5	4440.9±974.01	4557.54±1061.94	2.26±2.27	566.5±308.65	3.69±0.94	105.79±54.59
	MMAE	-	20.03±6.62	0.52±0.108	26.90±5.22	-	-	-	-	-
RC48-ADC Ab 8 mg kg^-1^	RC48-ADC Ab	185.93±56.33	0.02±0.00	100.55±12.81	4603.37±654.59	5170.36±896.82	10.45±6.12	428.95±176.88	1.59±0.29	276.85±54.99
MMAE 0.16 mg kg^-1^	MMAE	-	0.017±0.00	227.67±103.11	158.06±56.74	-	-	-	-	-

Note: ^a^Parameter unit of MMAE was ng mL^-1^; ^b^Parameter unit of MMAE was h·ng mL^-1^. *C*_max_ - maximum concentration, the highest concentration of a drug achieved in the bloodstream or a specific tissue after administration. AUC_(0- t)_ - area under the concentration-time curve from time 0 to time *t*; AUC_(0-inf)_ - Area under the concentration-time curve from time 0 to infinity); *V*_d_ - Volume of distribution, a theoretical volume that relates the amount of drug in the body to the concentration of the drug in the bloodstream.

FM showed linear pharmacokinetics in the dose range of 4-8 mg/kg and nonlinear pharmacokinetics when the dose was increased to 16 mg/kg (Figure 2 and Table 1). Compared with the same dose of the RC48-ADC group (8 mg/kg), the Tab concentration of the combined group was similar to that of the RC48-ADC group (Figure 2 and Table 1). Meanwhile, the plasma peak concentration and exposure levels of FM in the RC48-ADC group (8 mg/kg) were approximately 0.09 and 5.56 % of the equivalent directly injected combined group, respectively (Table 1).

The concentration-time curve of FM in the RC48-ADC group of rats showed a bimodal phenomenon (Figure 2). These in vivo results were strongly supported by our previous finding that only a small percentage of MMAE is released after the in vitro incubation of RC48-ADC with the serum of rats. According to these in vitro and in vivo results, the linker of RC48-ADC is expected to be stable in the human systemic circulation. Compared with the same dose of the RC48-ADC group, the plasma peak concentration and exposure levels of FM in the equal-quality combined group increased tremendously, which indicates that the molecular structure of RC48-ADC effectively reduces the exposure of small molecules in the body. This part of the experiment not only elucidates the pharmacokinetic characteristics of RC48-ADC in rats but also provides data support for the prediction of human PK by the allometric scaling method [13].

### Distribution

**Figure 3.**
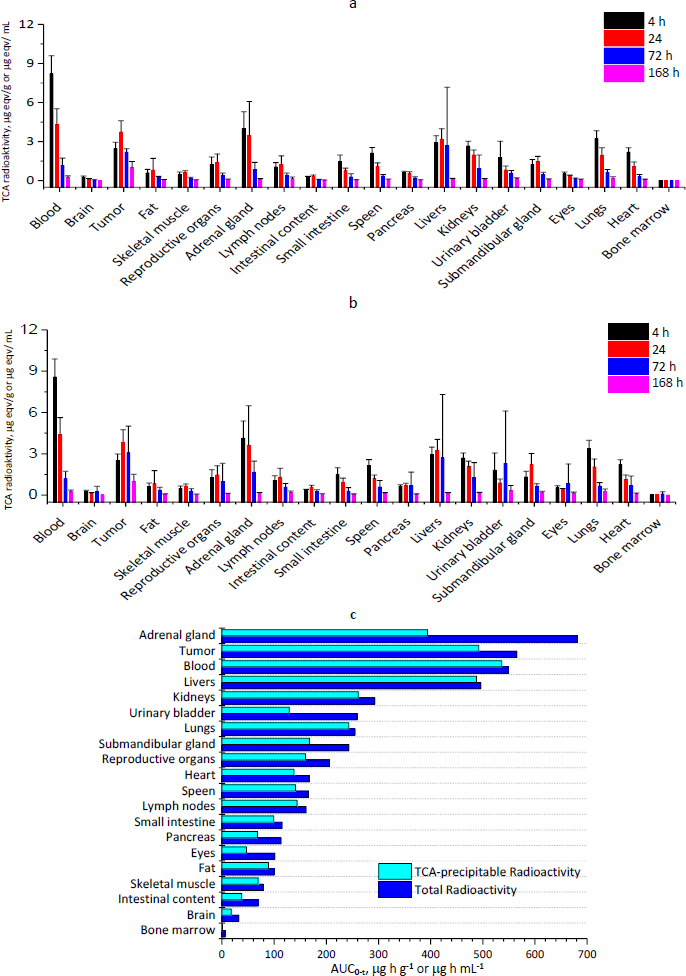
Tissue distribution of [^125^I]–RC48-ADC following a single i.v. bolus administration of 12 mg/kg (mean ± SD, *n* = 5 per time point per group). (a) Tissue concentrations of radioactivity by total radioactivity method; (b) Tissue concentrations of radioactivity by TCA-precipitable radioactivity method; (c) Tissue exposure (AUC_*0-t*_)

Overall, the distribution of the total radioactivity was similar to that of the TCA-precipitable radioactivity with the exception of a few tissues/body fluids (adrenal gland, urinary bladder) (Figure 3a, b). The exposure levels (AUCs) of the TCA-precipitable radioactivity in the tissues can be ranked from high to low in the following order: serum, tumour, liver, adrenal glands, kidneys, lungs, urine, submandibular gland, gonads, lymph nodes, spleen, heart, bladder, small intestine, adipose tissue, muscle, pancreas, eyeballs, intestinal faeces, intestinal contents, brain and bone marrow (Figure 3c). Conversely, the TCA-precipitable radioactivity in the blood accounted for approximately 90 % of the total radioactivity. The SHPLC analysis also showed that ^125^I–RC48-ADC is the main form that exists in the serum and no ^125^I–RC48-ADC and plasma protein conjugate or other metabolite was detected, which indicates that the drug exists mainly in its original form in the blood. Moreover, the tissues and organs rich in blood flow (such as the adrenal gland, liver, kidneys, and lungs) had higher radioactivity (Figure 3c). The radioactivity distribution was less in tissues and organs with poorer perfusion (such as muscle and adipose tissue). The relatively low radioactive concentration of the test article in the brain suggests that it does not pass the blood-brain barrier easily. It is worth noting that the radioactive concentration at the tumour site was relatively high, suggesting that RC48-ADC may possess excellent targeting.

Considering that the study on tissue distribution in tumour-bearing mice can better reflect the distribution of RC48-ADC in vivo and tumour targeting in the presence of tumour, the tissue distribution experiments in this study were carried out in tumour-bearing mice. From the tissue distribution study of ^125^I-RC48-ADC, we found that RC48-ADC was present in the blood mainly in the form of a prototype drug, and it's easier to distribute to the tissues and organs that are rich in blood flow and it's harder to cross the blood-brain barrier. The total radioactivity was very high, but the TCA-precipitable radioactivity was low in the urinary bladder, suggesting that a large amount of ^125^I RC48-ADC was degraded in the adrenal gland. The acid-precipitable radioactivity of ^125^I-RC48-ADC in the bladder and kidneys was high, whereas it was very low in the small intestinal and intestinal contents, which means that metabolites of ^125^I-RC48-ADC may be present in urine. This was also confirmed in the excretion experiment. Last but not least, RC48-ADC shows excellent targeting capability with a very high radioactive concentration of ^125^I-RC48-ADC at the tumour site, either the total or TCA-precipitable radioactivity.

**Figure 4.**
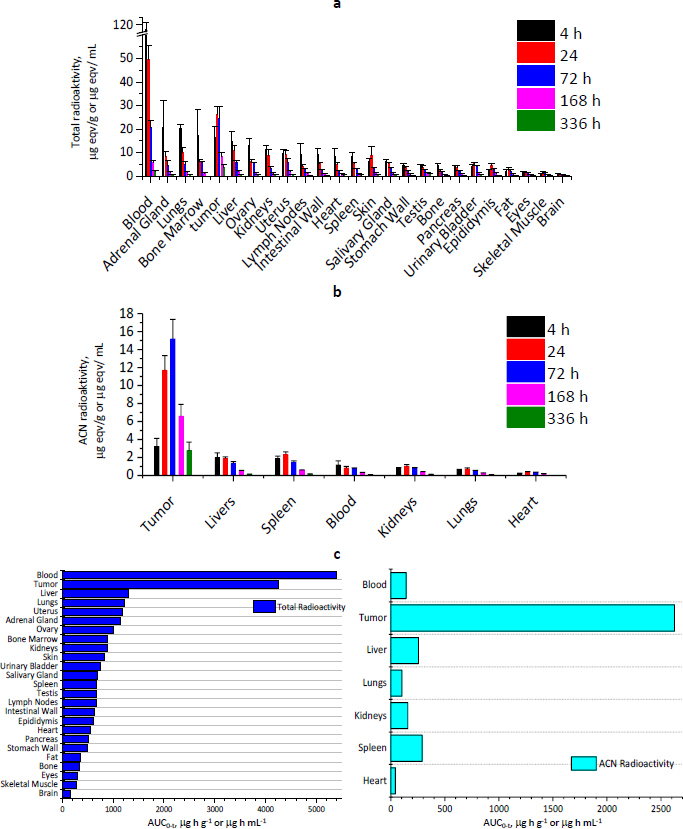
Tissue distribution of [^3^H-MMAE]-RC48-ADC following a single i.v. dose of 12 mg/kg (Mean ± SD, *n* =6 per time point per group). (a) Tissue concentrations of radioactivity by total radioactivity method; (b) Tissue concentrations of radioactivity by ACN-extract Radioactivity method; (c) Tissue exposure (AUC_0-t_)

The total and ACN-extract fraction radioactivity results of radioactive concentrations at various time points (Figure 4a, b) showed that with time, the FM in plasma decreased, while it in tissues and tumours increased significantly, the FM in the spleen, kidney, lung, and heart peaked at 24 hours, and the FM in tumours peaked at 72 hours. In other tissues and tumours, it peaked at 4 hours and 24 hours, respectively, which were lower than the same time point in plasma, and the peak concentration of radioactivity in the tumour was about 52.67 % of the same time point in plasma. In addition, the tumour radiation concentration at the last collection point (336 h) was the highest, about 1.93 times the plasma concentration at the same time point.

Meanwhile, according to the total radioactivity AUC_0-t_ from high to low tissues/fluids were the tumours, whole blood, liver, lung, uterus, adrenal gland, ovary, bone marrow, kidney, skin, bladder, submandibular gland, spleen, testis, lymph nodes, intestinal wall, epididymis, heart, pancreas, gastric wall, body fat, bone, eye, skeletal muscle, and brain (Figure 4c). The ACN-extract fraction radioactivity AUC_0-t_ from high to low tissues/fluids were blood, the tumours, liver, lung, uterus, adrenal gland, ovary, bone marrow, kidney, skin, bladder, submandibular gland, spleen, testis, lymph nodes, intestinal wall, epididymis, heart, pancreas, gastric wall, body fat, bone, eye, skeletal muscle, and brain (Figure 4c). The ACN-extract fraction AUC_0-t_ results showed the AUC_0-t_ ratio of tissues and plasma extractable fraction in tumour, spleen, liver, and kidney from high to low, which were 18.57, 2.04, 1.81, and 1.11 times plasma, respectively (Figure 4c). The results suggested that MMAE and its metabolites were mainly distributed in tumours, spleen, liver, and kidney.

Judged from the tissue distribution study of [^3^H-MMAE]-RC48-ADC, the exposure of ^3^H-MMAE in the tumour was the highest and much higher than the plasma and several other tissues and exposure was more persistent. The results suggested that although RC48-ADC is highly exposed in plasma, most of the time, it exists in the form of RC48-ADC, while small molecular payloads are rarely released in plasma, the vast majority of ^3^H-MMAE was released by RC48-ADC in the tumour location. These above outcomes indicated that MMAE and its metabolites were mainly distributed in tumours, spleen, liver, and kidney, and RC48-ADC showed excellent performance of tumour targeting, but the toxicity of the spleen, liver, and kidney also should be paid attention to.

### Excretion

**Figure 5.**
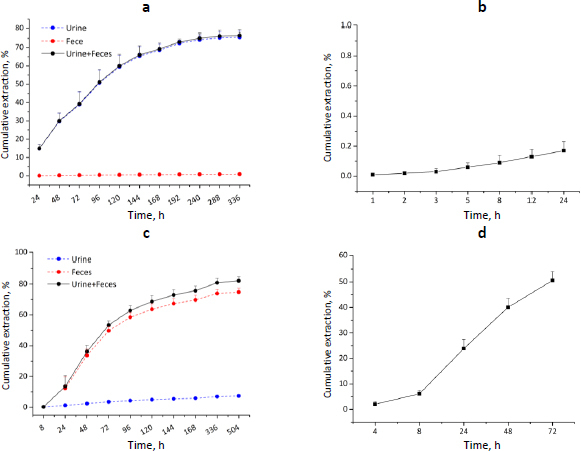
Excretion of [^125^I]–RC48-ADC and [^3^H-MMAE] -RC48-ADC in urine, faeces, and bile. (a) Urine and faeces excretion of [^125^I]–RC48-ADC in tumour-bearing nude mice following a single bolus i.v. administration of 12 mg/kg (Mean ± SD, *n* = 12 per time point per group); (b) Bile excretion of [^125^I]–RC48-ADC in the rats following a single bolus *i.v.* administration of 8 mg/kg (Mean ± SD, *n* = 6 per time point per group); (c) Urine and faeces excretion of [^3^H-MMAE] -RC48-ADC in the SD rats following a single bolus i.v. administration of 8 mg/kg (Mean ± SD, *n* = 6 per time point per group,); (d) Bile excretion of [^3^H-MMAE] -RC48-ADC in the SD rats following a single bolus i.v. administration of 8 mg/kg (Mean ± SD, *n* = 6 per time point per group)

The excretion of the RC48-ADC was relatively slow and primarily in the urine (Figure 5a).

After 336 h, the excretion in the urine and the faeces accounted for 75.64±2.80 and 0.90±0.13 % of the administered radioactivity, respectively. The SHPLC analysis showed that only small-molecule degradation products were present in the urine, not the original form of the test article. The 24-hour cumulative excretion in the bile accounted for 0.17±0.06 % of the administered radioactivity (Figure 5b). The excretion of RC48-ADC radioactivity was relatively slow and primarily in the urine, meanwhile, the faeces and bile were bare of RC48-ADC radioactivity. These results are consistent with the findings in the tissue distribution study that the radioactive concentration of RC48-ADC in the bladder and kidneys is much higher than in the small intestine and intestinal contents.

Furthermore, the urine and faeces excretion of [3H-MMAE]-RC48-ADC in rats was shown in Figure 5c. The total urine and faeces excretion of MMAE accounted for 9.17 and 74.69 % of the total dosage, respectively. Excretion of MMAE mainly occurred in the first week, accounting for 77.11 % of the drug dosage. The bile secretion of MMAE in bile-duct-cannulated (BDC) rats accounted for 78.44 % of the total 0-72 h excretion, suggesting that biliary faecal route excretion was the main excretion route of MMAE (Figure 5c,d).

### Metabolite

**Figure 6.**
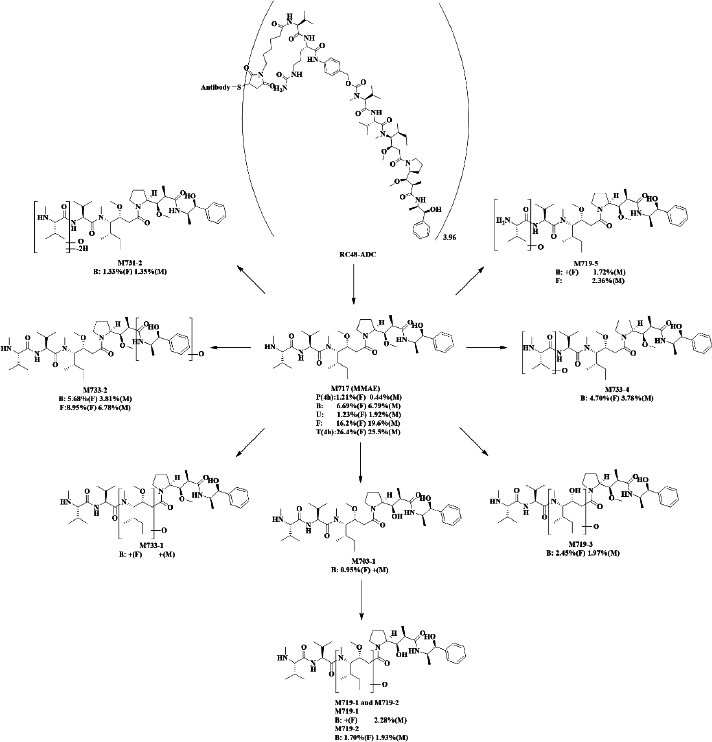
Major metabolic pathways of [^3^H]-labelled disitamab vedotin in rats/mice. P: plasma, represented by rate, % in B: bile, U: urine, F: faeces, representing and stool, respectively, represented by dose, %; T: tumour tissue, represented by rate, % of F: female and M: male rats with tumour tissue

Because large molecular proteins could not be extracted by organic solvent and remained in the extraction residue, only the payload (MMAE) of RC48-ADC and its metabolites were analysed in this study. As shown in Table 2, 11 catabolites were identified, including MMAE(M717), demethylation, and mono-oxidation metabolites (M719-1, M719-2, M719-3, M719-4, and M719-5), mono-oxidation metabolites (M733-1, M733-2, and M733-4), Demethylation metabolite (M703-1), Oxidative dehydrogenation metabolite (M731-2). MMAE prototype was detected in bile, urine, fences, plasma, and tumour tissue, all 10 metabolites of MMAE were detected in bile, and M719-5, M733-2, and M703-1 were also detected in faeces. The main metabolic pathways and metabolites of [^3^H-MMAE]RC48-ADC in rats and tumour-bearing mice are shown in Figure 6.

**Table 2.** Metabolite assignments and proposed metabolic transformation and structure for 11 metabolites observed in the excretion study.

No.	Catabolites	Protonated molecular formula	*m*/*z* for [M+H]+		Mass accuracy, ppm	Origin
			Theoretical	Measured		
1	M717 (MMAE)	C_39_H_68_N_5_O_7_+	718.5113	718.5100	-1.30	plasma/bile/urine/ /faeces/tumour tissue
2	M719-1	C_38_H_66_N_5_O_8_+	720.4906	720.4895	-1.10	bile
3	M719-2	C_38_H_66_N_5_O_8_+	720.4906	720.4900	-0.60	bile
4	M719-3	C_38_H_66_N_5_O_8_+	720.4906	720.4891	-1.50	bile
5	M719-4	C_38_H_66_N_5_O_8_+	720.4906	720.4896	-1.00	bile
6	M719-5	C_38_H_66_N_5_O_8_+	720.4906	720.4895	-1.10	bile/faeces
7	M733-1	C_38_H_68_N_5_O_8_+	734.5062	734.5051	-1.10	bile
8	M733-2	C_38_H_68_N_5_O_8_+	734.5062	734.5048	-1.40	bile/faeces
9	M733-4	C_38_H_68_N_5_O_8_+	734.5062	734.5053	-0.90	bile
10	M703-1	C_38_H_66_N_5_O_7_+	704.4957	704.4943	-1.40	bile/faeces
11	M731-2	C_39_H_66_N_5_O_8_+	732.4906	732.4887	-1.90	bile

In the collected bile and urine samples, eight catabolites, including MMAE of the polatuzumab vedotin (POLIVY^®^), were observed [14]. For brentuximab vedotin, twelve metabolites were identified in vitro in different species of hepatocyte metabolites identification experiments[13]. According to the results of the metabolites study, a total of 11 metabolites were identified, and the MMAE prototype was detected in bile, urine, faeces, plasma, and tumour tissue. All 10 metabolites of MMAE were detected in bile, and M719-5, M733-2 and M703-1 were also detected in faeces. This is further evidence that RC48-ADC was mainly eliminated through renal excretion. The elimination of [^3^H]-MMAE-RC48-ADC was mainly through biliary faecal secretion, and this claim was also approved when we characterized the catabolites found in urine and bile. Yip, Victor *et al.* [14] have reported that the elimination of [^3^H]-MMAE-polatuzumab vedotin in rats was mainly through a faecal–biliary path. Meanwhile, Han *et al.* [15] also found that patients with CD30-positive hematologic malignancies were given brentuximab vedotin, the primary excretion route of MMAE is via faeces and most in prototype form, which corroborated with our result as polatuzumab vedotin and brentuximab vedotin utilized the same linker and payload asRC48-ADC. Moreover, we also found that the average total radiation recovery rate (64.46 %) of biliary duct intubation rats was higher than that of normal rats (54.33 %) at the same time, suggesting the possibility of hepatoenteral circulation.

## Conclusion

In summary, the results showed good plasma pharmacokinetics and excellent targeting to tumours for disitamab edotin and effective detoxification during hepatobiliary/bile elimination for disitamab vedotin is consistent with its favourable safety profiles. Continued understanding of the ADME properties of disitamab vedotin should provide more theoretical basis and data support for clinical and additional insight into what attributes may be necessary for better clinical application. To better understand or predict the characteristics of ADME in vivo of RC48-ADC, the best efficacy and safety when applied to clinical patients must be achieved. Hot and emerging applications such as drug-drug interaction (DDI) (predicting the safety of combination with small molecules or biomacromolecules) and PK/PD modelling (predicting/optimizing clinical dose scheme design) deserve more attention and application.

## References

[1] Hafeez U. Parakh S. Gan H.K. Scott A.M. Antibody-Drug Conjugates for Cancer Therapy. Molecules 25 (2020) 4764. https://doi.org/10.3390/molecules25204764 10.3390/molecules25204764 33081383 PMC7587605

[2] do Pazo C. Nawaz K. Webster R.M. The oncology market for antibody-drug conjugates. Nat. Rev. Drug Discovery 20 (2021) 583-584. https://doi.org/10.1038/d41573-021-00054-2 10.1038/d41573-021-00054-2 33762691

[3] Jiang J. Dong L. Wang L. Wang L. Zhang J. Chen F. Zhang X. Huang M. Li S. Ma W. Xu Q. Huang C. Fang J. Wang C. HER2-targeted antibody drug conjugates for ovarian cancer therapy. European Journal of Pharmaceutical Sciences 93 (2016) 274-286. https://doi.org/10.1016/j.ejps.2016.08.015 10.1016/j.ejps.2016.08.015 27509865

[4] Sheng X. Yan X. Wang L. Shi Y. Yao X. Luo H. Shi B. Liu J. He Z. Yu G. Ying J. Han W. Hu C. Ling Y. Chi Z. Cui C. Si L. Fang J. Zhou A. Guo J. Open-label, Multicenter, Phase II Study of RC48-ADC, a HER2-Targeting Antibody-Drug Conjugate, in Patients with Locally Advanced or Metastatic Urothelial Carcinoma. Clinical Cancer Research 27 (2021) 43-51. https://doi.org/10.1158/1078-0432.CCR-20-2488 10.1158/1078-0432.CCR-20-2488 33109737

[5] Peng Z. Liu T. Wei J. Wang A. He Y. Yang L. Zhang X. Fan N. Luo S. Li Z. Gu K. Lu J. Xu J. Fan Q. Xu R. Zhang L. Li E. Sun Y. Yu G. Bai C. Liu Y. Zeng J. Ying J. Liang X. Xu N. Gao C. Shu Y. Ma D. Dai G. Li S. Deng T. Cui Y. Fang J. Ba Y. Shen L. Efficacy and safety of a novel anti-HER2 therapeutic antibody RC48 in patients with HER2-overexpressing, locally advanced or metastatic gastric or gastroesophageal junction cancer: a single-arm phase II study. Cancer Communications 41 (2021) 1173-1182. https://doi.org/10.1002/cac2.12214 10.1002/cac2.12214 34665942 PMC8626607

[6] Xu Y. Wang Y. Gong J. Zhang X. Peng Z. Sheng X. Mao C. Fan Q. Bai Y. Ba Y. Jiang D. Yang F. Qi C. Li J. Wang X. Zhou J. Lu M. Cao Y. Yuan J. Liu D. Wang Z. Fang J. Shen L. Phase I study of the recombinant humanized anti-HER2 monoclonal antibody-MMAE conjugate RC48-ADC in patients with HER2-positive advanced solid tumors. Gastric Cancer 24 (2021) 913-925. https://doi.org/10.1007/s10120-021-01168-7 10.1007/s10120-021-01168-7 33945049 PMC8205919

[7] Kalmuk J. Rinder D. Heltzel C. Lockhart A.C. An overview of the preclinical discovery and development of trastuzumab deruxtecan: a novel gastric cancer therapeutic. Expert Opinion on Drug Discovery 17 (2022) 427-436. https://doi.org/10.1080/17460441.2022.2050692 10.1080/17460441.2022.2050692 35426752

[8] Li C. Zhang C. Deng R. Leipold D. Li D. Latifi B. Gao Y. Zhang C. Li Z. Miles D. Chen S.C. Samineni D. Wang B. Agarwal P. Lu D. Prabhu S. Girish S. Kamath A.V. Prediction of Human Pharmacokinetics of Antibody-Drug Conjugates from Nonclinical Data. Clinical and Translational Science 12 (2019) 534-544. https://doi.org/10.1111/cts.12649 10.1111/cts.12649 31115997 PMC6742937

[9] Liu S.N. Li C. Clinical pharmacology strategies in supporting drug development and approval of antibody-drug conjugates in oncology. Cancer Chemotherapy and Pharmacology 87 (2021) 743-765. https://doi.org/10.1007/s00280-021-04250-0 10.1007/s00280-021-04250-0 33792763 PMC8110483

[10] Drake `P.M. Rabuka D. Recent Developments in ADC Technology: Preclinical Studies Signal Future Clinical Trends. BioDrugs 31 (2017) 521-531. https://doi.org/10.1007/s40259-017-0254-1 10.1007/s40259-017-0254-1 29119409 PMC5696438

[11] Kamath A.V. Iyer S. Challenges and advances in the assessment of the disposition of antibody-drug conjugates. Biopharm. Drug Dispos. 37 (2016) 66-74. https://doi.org/10.1002/bdd.1957 10.1002/bdd.1957 25904406 PMC5032988

[12] Bolleddula J. Brady K. Bruin G. Lee A. Martin J.A. Walles M. Xu K. Yang T.Y. Zhu X. Yu H. Absorption, Distribution, Metabolism, and Excretion of Therapeutic Proteins: Current Industry Practices and Future Perspectives. Drug Metabolism and Disposition 50 (2022) 837-845. https://doi.org/10.1124/dmd.121.000461 10.1124/dmd.121.000461 35149541

[13] The U.S. Food and drug administration. https://www.accessdata.fda.gov/drugsatfda_docs/nda/2011/125388Orig1s000PharmR.pdf 10.1080/15360280801989401 28792814

[14] Yip V. Lee M.V. Saad O.M. Ma S. Khojasteh S.C. Shen B.Q. Preclinical Characterization of the Distribution, Catabolism, and Elimination of a Polatuzumab Vedotin-Piiq (POLIVY^®^) Antibody-Drug Conjugate in Sprague Dawley Rats. Journal of Clinical Medicine 10 (2021) 1323. https://doi.org/10.3390/jcm10061323 10.3390/jcm10061323 33806916 PMC8004598

[15] Han T.H. Gopal A.K. Ramchandren R. Goy A. Chen R. Matous J.V. Cooper M. Grove L.E. Alley S.C. Lynch C.M. O'Connor O.A. CYP3A-mediated drug-drug interaction potential and excretion of brentuximab vedotin, an antibody-drug conjugate, in patients with CD30-positive hematologic malignancies. The Journal of Clinical Pharmacology 53 (2013) 866-877. https://doi.org/10.1002/jcph.116 10.1002/jcph.116 23754575 PMC3777854

